# Influence of the Surface Container Nature on the Magnetic Properties of a Magnetic Eutectic Mixture

**DOI:** 10.1002/cphc.202500529

**Published:** 2025-10-28

**Authors:** Cristina Zapater, Miguel Ángel Aguirre, Lorena Vidal, Antonio Canals

**Affiliations:** ^1^ Department of Analytical Chemistry Nutrition and Food Science and University Institute of Materials Faculty of Science University of Alicante P.O. Box 99 03080 Alicante Spain

**Keywords:** glass materials, magnetic eutectic mixtures, magnetic properties, polypropylene materials

## Abstract

The effect of polypropylene and glass materials on the magnetic properties of a magnetic eutectic mixture (MEM) composed of iron chloride hexahydrate (FeCl_3_·6H_2_O) and ethylene glycol (EG) (i.e., FeCl_3_·6H_2_O:EG, molar ratio of 1:2) has been investigated. In both materials, magnetization (M) curves at 300 and 358 K and effect of temperature are studied. As a result, MEM exhibits paramagnetic behavior. However, MEM on polypropylene exhibits a higher slope of the linear fit at 300 K (i.e., 4.03 × 10^−6^ emu g^−1^ Oe^−1^) compared to 358 K (i.e., 1.90 × 10^−6^ emu g^−1^ Oe^−1^) and a decrease of its M with increasing temperature. Conversely, MEM on glass exhibits a lower slope of the linear fit at 300 K (i.e., 4.05 × 10^−6^ emu g^−1^ Oe^−1^) compared to 358 K (i.e., 4.55 × 10^−6^ emu g^−1^ Oe^−1^), remaining practically stable at higher temperatures. Notably, magnetization on polypropylene (i.e., 4.46 × 10^−2^ emu g^−1^) is higher at room temperature than that of glass (i.e., 3.86 × 10^−2^ emu g^−1^), whereas at synthesis temperature, glass shows a higher magnetization (i.e., 3.75 × 10^−2^ emu g^−1^) compared to polypropylene (i.e., 3.43 × 10^−2^ emu g^−1^). These findings underscore the influence of surface containers on the magnetic properties of MEM, which should be considered when selecting materials for specific chemical applications.

## Graphical abstract

The nature of the contact surface material affects the magnetic properties of a magnetic eutectic mixture (MEM). In polypropylene, MEM maintains its chemical structure and magnetism , whereas on glass surfaces, its chemical structures could be altered, increasing its adhesion to the surface and losing magnetism. These findings emphasize the importance of selecting suitable container material in MEM‐based chemical applications.

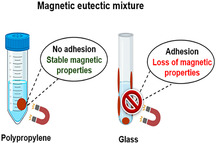

## 1 Introduction

In the search for increasingly environmentally friendly solvents, there has been a growing interest in eutectic mixtures, commonly known as deep eutectic solvents (DESs), due to their simple synthesis and interesting physicochemical properties such as low melting point, thermal stability, low vapor pressure, and biodegradability, among others.^[^ ^1^, ^2^ ^]^ In 2017, Khezeli and Daneshfar^[^ ^3^ ^]^ described for the first time a new subtype of eutectic mixture, commonly known as magnetic deep eutectic solvents (MDESs), which are obtained by mixing a hydrogen bond acceptor (HBA), a hydrogen bond donor (HBD), and a metal component at elevated temperatures (i.e., 80–100 °C) resulting in ternary MDESs. Subsequently, Shi et al.^[^ ^4^ ^]^ characterized a binary MDES constituted exclusively by the HBD and the metal component, which behaves as the HBA. The terms of DESs and MDESs are currently under scientific debate because the eutectic point temperature is not always lower than that of an ideal liquid mixture.^[^ ^5^ ^]^ Therefore, in this study, the generic term magnetic eutectic mixtures (MEMs) is employed.

MEMs have promising properties that make them very interesting in different applications.^[^ ^6^, ^7^ ^]^ For example, their use in electrochemistry has been described due to their high ionic conductivity,^[^ ^8^ ^]^ as well as their use as heterogeneous catalysts in organic synthesis.^[^ ^9^ ^]^ In addition, their magnetic properties allow their use as extractant solvents facilitating their manipulation and recovery with an external magnetic field, thus reducing the time and energy consumption by not requiring a centrifugation step.^[^ ^10^ ^]^ Thereby, in the scientific literature, MEMs have been used as extractants to separate and preconcentrate DNA from biological samples,^[^ ^11^ ^]^ various organic compounds from biological, environmental, and food samples,^[^ ^12^, ^13^, ^14^, ^15^, ^16^ ^]^ and for the extraction of inorganic analytes from water and food samples.^[^ ^17^ ^]^

On the other hand, some works have also focused on the study of their physicochemical properties, such as their viscosity, conductivity, surface tension, thermal stability, and magnetic properties.^[^ ^4^, ^18^ ^]^ One of our recent studies investigates the different behavior of MEMs in contact with polypropylene and glass materials. Specifically, the study demonstrated the adhesion of MEMs to glass surfaces and the subsequent inability to manipulate them using an external magnetic field such as a magnet. In contrast, MEMs in polypropylene showed a distinct behavior with the absence of adhesion and consequently responding to external magnetic fields.^[^ ^19^ ^]^ In addition, different behaviors were visually observed in response to the action of an external magnetic field depending on the material in contact with the MEM. These findings could have important implications in some chemical processes such as liquid–liquid (micro)extraction.^[^ ^20^ ^]^ Therefore, it is important to understand the fundamental reasons behind the different magnetic behavior of these mixtures depending on the material in contact with them.

To the best of our knowledge, no research has investigated the effect that the nature of the surface container can have on the magnetic properties of MEMs. Therefore, this work investigates the magnetization (M) of a MEM composed of iron chloride hexahydrate (FeCl_3_·6H_2_O) and ethylene glycol (EG) in a molar ratio of 1:2 (i.e., FeCl_3_·6H_2_O:EG) in contact with polypropylene and glass materials. Specifically, magnetization was studied as a function of applied magnetic fields ranging from −50 000 Oe to +50 000 Oe at room temperature (i.e., 300 K) and at synthesis temperature (i.e., 358 K), and at different temperatures (i.e., 273, 300, 328, and 358 K) by applying an external magnetic field of 1000 Oe.

## 2 Results and Discussion

### 2.1 Magnetization Curves

**Figure 1:**
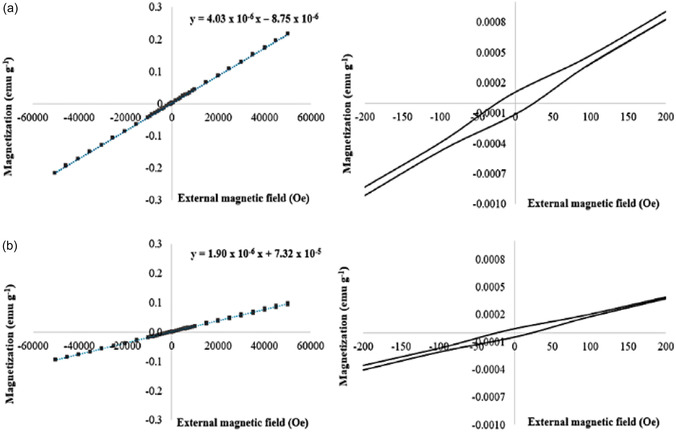
Magnetization curves of MEM (i.e., FeCl_3_·6H_2_O:EG, 1:2) on polypropylene material at a) 300 K and b) 358 K. The right‐side graphs represent an enlarged scale of the magnetic field for better visualization.

**Figure 2:**
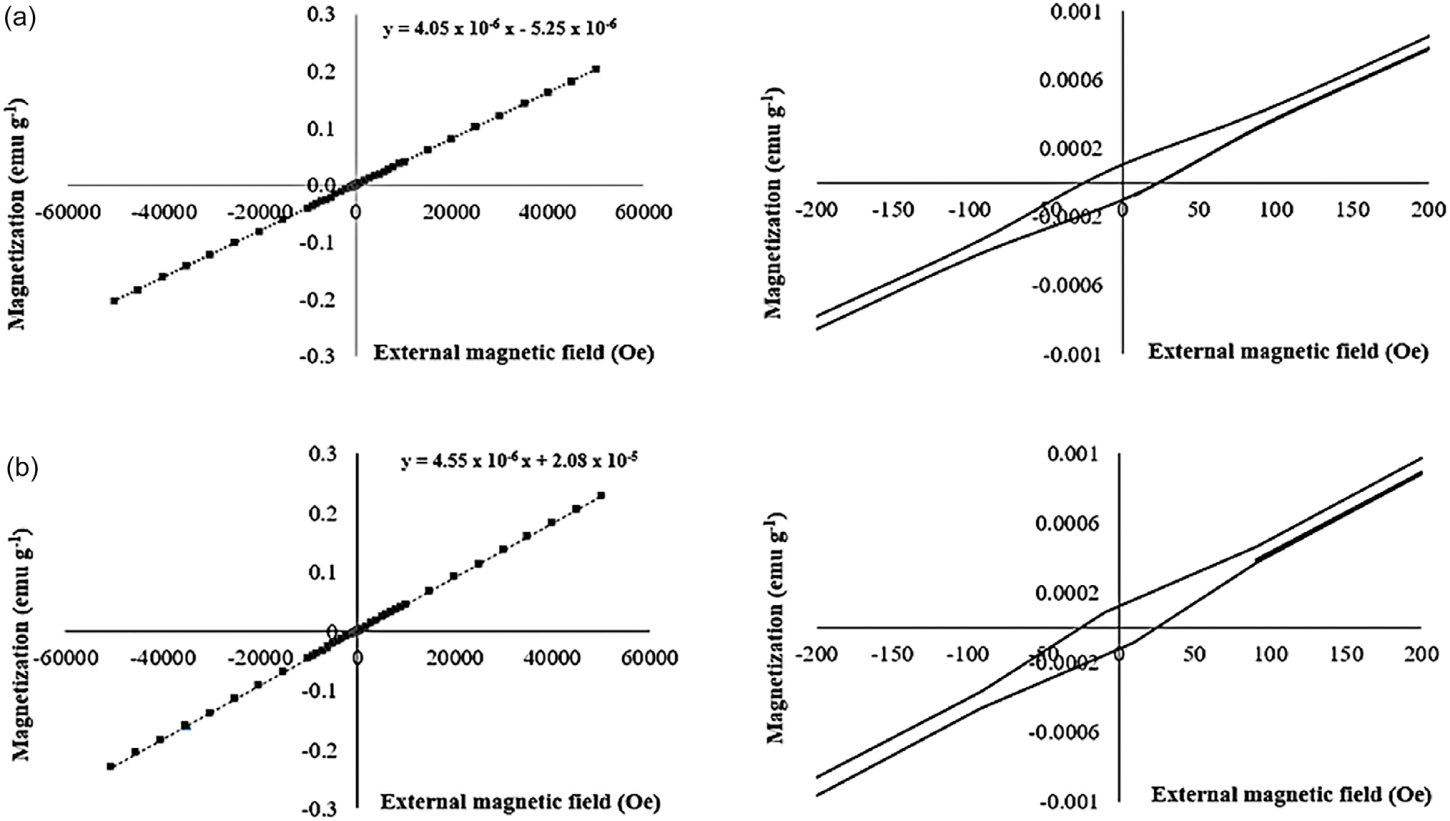
Magnetization curves of MEM (i.e., FeCl_3_·6H_2_O:EG, 1:2) on glass material at a) 300 K and b) 358 K. The right‐side graphs represent an enlarged scale of the magnetic field for better visualization.

The magnetization (M) of the MEM exhibited linear responses to applied external magnetic fields (H) in both polypropylene (**Figure** **1**) and glass materials (**Figure** **2**). It is well established that ferromagnetic materials manifest a hysteresis loop in their magnetization curves. Thus, the absence of a hysteresis loop in the MEM magnetization curves indicates its paramagnetic behavior.^[^ ^21^ ^]^ Specifically, the paramagnetic behavior is characterized by a transient and linear magnetization process that does not result in the retention of magnetization following the removal of an external magnetic field.^[^ ^22^ ^]^

However, it should be noted that the values of the slopes of the linear fits were different depending on the material of the container and temperature. Specifically, the MEM on polypropylene at 300 K exhibited a higher slope of the linear fit (i.e., 4.03 × 10^−6^ emu g^−1^ Oe^−1^) (Figure 1a) compared to the slope obtained at 358 K (i.e., 1.90 × 10^−6^ emu g^−1^ Oe^−1^ **)** (Figure 1b). These values are consistent with the principle of thermodynamics, which postulates that an increase in the temperature results in a decrease in the M.^[^ ^23^ ^]^ However, MEM on glass showed a lower slope of the linear fit at 300 K (i.e., 4.05 × 10^−6^ emu g^−1^ Oe^−1^) (Figure 2a) than at 358 K (i.e., 4.55 × 10^−6^ emu g^−1^ Oe^−1^) (Figure 2b). Consequently, MEM on glass material did not exhibit the expected behavior of a paramagnetic material.

### 2.2 Effect of the Temperature on Magnetization

**Figure 3:**
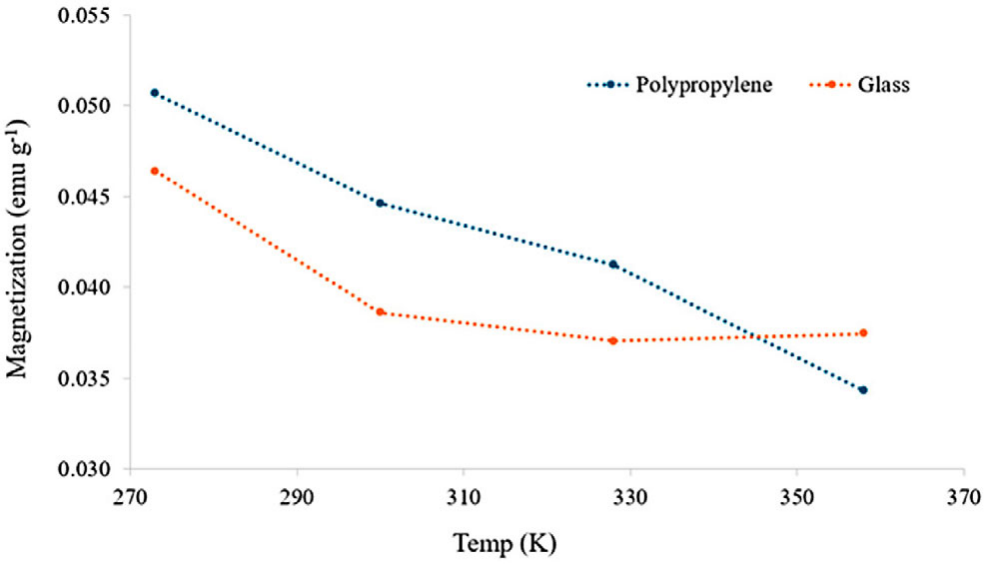
Magnetization (emu g^−1^) of MEM (i.e., FeCl_3_·6H_2_O:EG, 1:2) on polypropylene (blue line) and glass (orange line) at different temperatures (i.e., 273, 300, 328, and 358 K) applying an external magnetic field of 1000 Oe.

The study of the effect of the temperature on the magnetization of MEM revealed a distinct tendency depending on the type of surface container. Specifically, MEM on polypropylene exhibited a decrease of the magnetization (M) with increasing temperatures (M_273K_ > M_300K_ > M_328K_ > M_358K_), while on glass material it remained practically stable at elevated temperatures (M_273K_ > M_300K_ > M_328K_ ≤ M_358K_) (**Figure** **3**). Notably, MEM on polypropylene reached the highest magnetization value at the lowest temperature (i.e., 5.07 × 10^−2^ emu g^−1^ at 273 K) and the lowest magnetization value at the highest temperature (i.e., 3.43 × 10^−2^ emu g^−1^ at 358 K). Conversely, the magnetization of MEM on glass exhibited a discernible decline with rising temperature from 273 to 300 K. However, beyond this temperature, the decrease in MEM magnetization remained practically stable, even reaching a slightly higher magnetization at 358 K (i.e., 3.75 × 10^−2^ emu g^−1^) than at 328 K (i.e., 3.71 × 10^−2^ emu g^−1^). These results align with those previously reported by Yang et al.,^[^ ^21^ ^]^ who observed a decrease in magnetization as the temperature increased. In addition, it is noteworthy that the magnetization value of the MEM at 300 K on polypropylene (i.e., 4.46 × 10^−2^ emu g^−1^) was higher than that obtained on glass (i.e., 3.86 × 10^−2^ emu g^−1^), while at 358 K, the magnetization value was higher on glass (i.e., 3.75 × 10^−2^ emu g^−1^) than that on polypropylene (i.e., 3.43 × 10^−2^ emu g^−1^).

**Figure 4:**
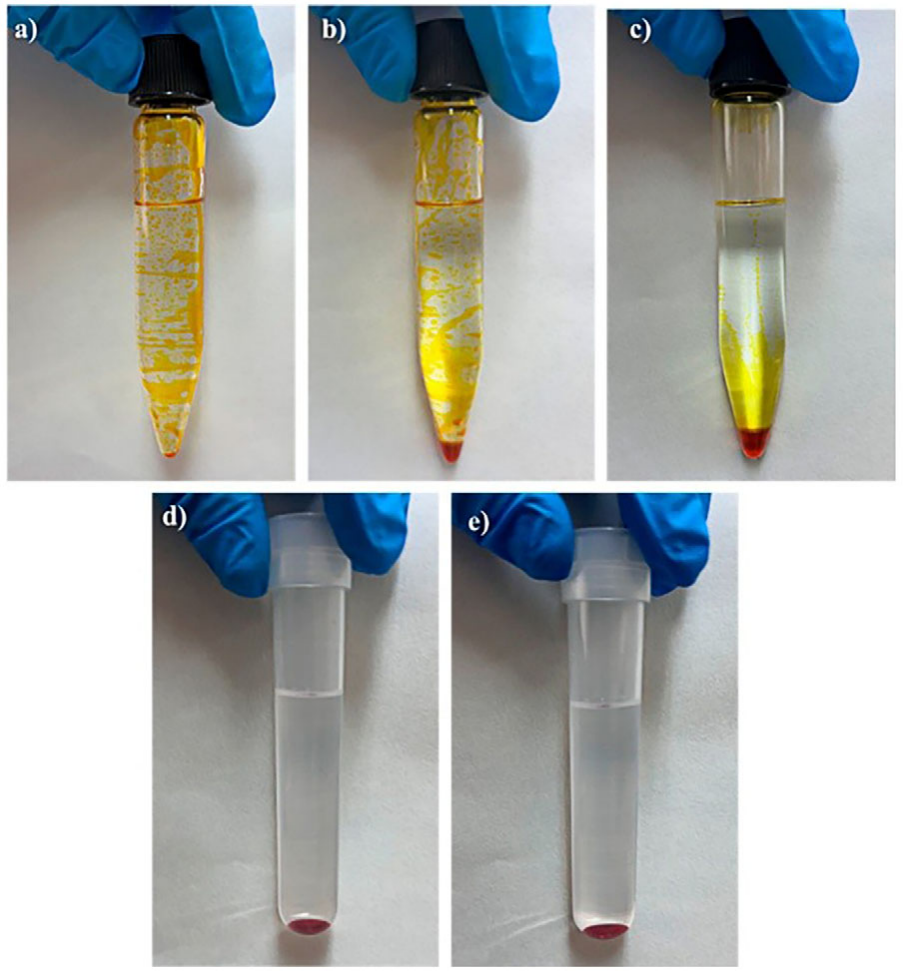
Images of MEM (i.e., FeCl_3_·6H_2_O:EG, 1:2) at ≈26 °C (300 K) in: a) glass tube after vortex agitation for 3.5 min. b) Glass tube after vortex agitation for 3.5 min and decanting 20 min. c) Glass tube after vortex agitation for 3.5 min and centrifugation for 3 min. d) Polypropylene tube after vortex agitation for 3.5 min. e) Polypropylene tube after vortex agitation for 3.5 min and centrifugation for 3 min.

These findings are consistent with those obtained in Section 2.1, and in both cases, they could be attributed to the adhesion of MEM to the surfaces of glass materials (**Figure** **4**a), which in turn would modify its magnetic properties due to the restriction of the movement of its magnetic moments, resulting in a lower expected magnetization in contact with glass surfaces at room temperature.

Furthermore, the adhesion strength of MEM to glass surfaces was notable, as evidenced by its ability to remain attached to glass surfaces even after 20 min of decanting (Figure 4b) and after a centrifugation step (Figure 4c). A possible explanation for this phenomenon could be that MEM is a mixture of weak H‐bonding interactions between its constituents and the interactions with the solid surface are stronger probably due to H‐bond interactions with silanol groups (–OH) on the glass surface resulting in the breakdown of the MEM, as demonstrated in a glass deactivation process.^[^ ^19^ ^]^ Consequently, the alteration of the chemical composition of the MEM in glass surfaces exhibits an increased degree of adhesion and loss of its magnetic properties. These findings are also supported by the results reported by Yang et al.,^[^ ^21^ ^]^ who demonstrated that alterations in the chemical composition of nanocrystals modify their magnetic properties.

On the other hand, the interaction between MEM and the surface of the glass container may be a contributing factor to the loss of accuracy using DES‐based liquid–liquid (micro)extraction.^[^ ^20^ ^]^ In contrast, MEM demonstrated no adhesion to polypropylene after vortex agitation (Figure 4d) and a centrifugation step (Figure 4e), maintaining its magnetic properties.

In a recent study, the surface tension and wettability of MEM on glass and polypropylene surfaces have been investigated.^[^ ^19^ ^]^ The MEM exhibits a surface tension of 71.8 ± 0.1 mN m^−1^, notably higher than that reported in the literature for a similar MEM (i.e., FeCl_3_·6H_2_O:EG, 2:1; 57.0 mN m^−1^).^[^ ^24^ ^]^ This difference can be attributed mainly to the higher ethylene glycol content in our MEM, which increases the number of hydroxyl (‐OH) groups available for hydrogen bonding. Furthermore, contact angle measurements reveal a substrate‐dependent wettability, showing a higher contact angle on polypropylene than on glass surface, indicating poor wettability between the MEM and polypropylene material.^[^ ^19^ ^]^ These results align with the observed adhesion phenomena and the distinct magnetic properties of MEM on glass and polypropylene materials.

## 3 Conclusion

This investigation shows that in polypropylene and glass materials the magnetization curves of a MEM (i.e., FeCl_3_·6H_2_O:EG, 1:2) exhibit a linear trend without the presence of a hysteresis loop, thereby indicating its paramagnetic behavior. From our knowledge, it has been experimentally proved for the first time that the nature of the surface container influences the magnetic properties of the MEMs.

Specifically, at 300 and 358 K, the magnetization curve of MEM on polypropylene exhibited a lower slope of the linear fit at higher temperatures, while the slope of magnetization linear fit on glass material was practically invariable. These results were consistent with those obtained in the study of the effect of temperature, where the magnetization of MEM on polypropylene decreased with increasing temperature, while its magnetization on glass material remained practically stable at elevated temperatures. Furthermore, it was noteworthy that at room temperature, the MEM exhibited higher magnetization on polypropylene, while at synthesis temperature, the magnetization was higher in contact with glass material.

These results are consistent with the adhesion phenomenon of MEM to glass surfaces, which could be attributed to the chemical interaction between the MEM and the chemical compounds of the glass surface container, resulting in a breakdown of its chemical structure and loss of magnetic properties. In contrast, the higher magnetization of MEM on polypropylene at room temperature could be due to the stability of its chemical structure, which results in its magnetic properties remaining intact and no adhesion phenomenon. Therefore, these findings highlight the influence of surface container nature on the magnetic properties of the MEM (i.e., FeCl_3_·6H_2_O:EG, 1:2) and underscore the importance of appropriately selecting the material type for the specific desired MEM‐based chemical application.

## 4 Experimental Section

4.1

4.1.1

##### Reagents and Materials

EG (purity 99.8%) was purchased from Honeywell (Charlotte, NC, USA) while FeCl_3_·6H_2_O (purity ≥ 98%) was obtained from Sigma Aldrich (St. Louis, MO, USA). The synthesis of MEM was carried out in a Schlenk flask (Scharlau, Barcelona, Spain) under static nitrogen gas (Carburos Metálicos, Barcelona, Spain) using a hotplate and a magnetic stirrer from J.P. Selecta (Barcelona, Spain). The resulting magnetic mixture was stored at room temperature in a desiccator from Normax (Marinha Grande, Portugal).

Magnetic measurements were carried out using openable polypropylene and glass capsules (2.4 × 1.05 cm, inner diameter of 0.85 cm). The polypropylene capsules were provided by Cangsheng Plastic Products Co., Ltd (Cangzhou, China) while the glass capsules were obtained from the glassblowing workshop of the Research Technical Services of the University of Alicante.

##### Apparatus and Instrumentation

Magnetization measurements were carried out using a Quantum Design MPMS‐XL‐5 SQUID magnetometer (San Diego, CA, USA). This equipment facilitates the measurements under both alternating current (AC) and direct current (DC) conditions by applying magnetic fields up to +\− 5 T. In this research, the magnetization curves were performed by AC conditions varying the magnetic field in a range from −50 000 Oe to +50 000 Oe at room temperature (i.e., 300 K), and at synthesis temperature (i.e., 358 K). To study the effect of temperature, it was investigated under DC conditions with a constant magnetic field of 1000 Oe at four different temperatures, including 273, 300, 328, and 358 K. Both sets of experiments were performed with 0.1 g of MEM on polypropylene and glass capsules. It is noteworthy that the diamagnetic contribution of plastic materials is negligible in the order of 1 × 10^−5^ emu at room temperature, while glass materials have no net contribution due to the fact that the sensor reads a constant signal.

##### Synthesis of Hydrophilic MEM

The synthesis of hydrophilic MEM consisted of mixing FeCl_3_·6H_2_O (0.04 mol) and EG (0.08 mol) (molar ratio 1:2) in a Schlenk flask under a static nitrogenatmosphere. The flask was subsequently sealed to maintain inert conditions at 85 °C (358 K), with continuous stirring for a duration of 30 min. This process resulted in a homogeneous brown liquid mixture, which should be stored in a desiccator to avoid moisture absorption.

## Conflict of Interest

The authors declare no conflict of interest.

## Author Contributions

**Cristina Zapater**: formal analysis (lead); investigation (lead); methodology (equal); validation (lead); visualization (lead); writing—original draft (lead); writing—review & editing (lead). **Miguel Ángel Aguirre**: conceptualization (equal); investigation (equal); methodology (equal); project administration (equal); supervision (lead); visualization (equal); writing—review & editing (equal). **Lorena Vidal**: conceptualization (equal); funding acquisition (lead); investigation (equal); project administration (lead); resources (lead); supervision (equal); visualization (equal); writing—review & editing (equal). **Antonio Canals**: conceptualization (lead); funding acquisition (lead); investigation (lead); project administration (lead); resources (lead); supervision (lead); visualization (equal); writing—review & editing (equal).

## Data Availability

The data that support the findings of this study are available from the corresponding author upon reasonable request.
